# Substrate scent‐induced disproportionate seed dispersal by rodents

**DOI:** 10.1002/ece3.70075

**Published:** 2024-07-21

**Authors:** Shuhua Guo, Xianfeng Yi, Meixia Sui

**Affiliations:** ^1^ College of Biology and Oceanography Weifang University Weifang China; ^2^ School of Life Sciences Qufu Normal University Qufu China

**Keywords:** leaf litter, odor masking, scatter hoarding, seed dispersal, seed odor

## Abstract

Conspecific adults impose strong negative density‐dependent effects on seed survival nearby parent trees, however, the underlying mechanisms are diversified and remain unclear. In this study, we presented consistent evidence that parent‐scented forest floor masked seed odor, reduced cache recovery rate by scatter‐hoarding animals, and then increased seed dispersal far away from mother trees. Our results showed that seed odors of Korean pine *Pinus koraiensis* match well with the volatile profile of their forest floor. Moreover, scatter‐hoarding animals selectively transported *P. koraiensis* seeds toward the areas where seed odor was more contrasting against the background substrate, possibly due to the fact that accumulation of conspecific volatile compounds in caches hindered seed detection by scatter‐hoarding animals. Our study provides insight into the role of leaf litter in directing seed dispersal process, representing a novel mechanism by which *P. koraiensis* increases selection for seed dispersal far away from the parent tree.

## Graphical abstract

Conspecific adults impose strong negative density‐dependent effects on seed survival nearby parent trees, however, the underlying mechanisms are diversified and remain unclear. Our study provides insight into the role of leaf litter in directing seed dispersal process, representing a novel mechanism by which *P. koraiensis* increases selection for seed dispersal far away from the parent tree.

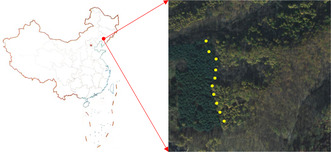

## 1 INTRODUCTION

Seed dispersal as well as seedling establishment play a critical role in shaping the patterns of seedling recruitment and spatial distribution of plant communities within many ecological systems (Meng et al., 2023; Steele et al., 2015; Vander Wall, 1990, 2010). Since the Janzen–Connell hypothesis was proposed (Connell, 1971; Janzen, 1970), to identify the underlying mechanisms that maintain species diversity, it has been a hotspot in plant community ecology (Chen, Feng, & Wang, 2022; Comita et al., 2014; Liang et al., 2016; Nathan et al., 2000; Pregitzer et al., 2010). The Janzen–Connell hypothesis predicts that adult trees can locally reduce the seedling recruitment of conspecifics through density‐dependent predation or infection by specific pathogens (Comita et al., 2014). Density‐dependent predation usually occurs when the seed survival rates vary causally with seed density. However, the possibilities of seed survival and the resulting seedling establishment patterns near or under canopy are quite variable (Chen, Chen, et al., 2022; Deniau et al., 2017; Liu et al., 2012; McCanny & Cavers, 1987), and may involve a number of factors, such as density‐dependent seed predation (Hirsch et al., 2012), microhabitat variability (Kadmon & Shmida, 1990), intra‐ and interspecific competition (Xiao et al., 2022; Yu et al., 2023), seedling herbivory (Packer & Clay, 2003), local pathogens (Freckleton & Lewis, 2006), and soil property (Pregitzer et al., 2010).

Negative conspecific interaction is of important significance for understanding coexistence of species in many communities (Chesson, 2000; Tilman, 1982). It is commonly expected that conspecific adult trees have widespread and strong negative effects on seed survival (Terborgh et al., 2002). Seed‐eating animals attracted by fruiting trees appear to be one of the important factors influencing seed survival and seedling establishment near parent trees. Hirsch et al. (2012) reported that scatter‐hoarding rodents tend to transport seeds towards areas with low conspecific tree density. Recently, Geng et al. (2018) presented similar evidence that scatter‐hoarding rodents moved seeds from seed sources to the area with low seed density in the experimental enclosures. Scatter‐hoarding rodents even favor cache locations where predation risk is high, increasing the possibility of cache survival (Mason et al., 2022; Steele et al., 2015). Although the underlying mechanism is not clear, directed seed dispersal by scatter‐hoarding animals benefits seed survival and seedling establishment far from the parent trees.

Scatter‐hoarding animals rely on both spatial memory and olfaction to locate buried seeds; however, olfaction appears to be crucial for scatter‐hoarding animals during foraging (Wang et al., 2018; Yi et al., 2021). Volatile molecules emitted from seeds act as key olfactory cues to facilitate foraging of scatter‐hoarding animals (Wang & Yi, 2022). Scatter‐hoarding animals use their sense of smell to locate seeds, including those that are buried in soil and plant litter and those cached by food‐hoarding animals (Dimitri & Longland, 2022; Vander Wall, 2010). Moreover, seed odor has been shown to influence seed caching of scatter‐hoarding animals (Yi, Li, et al., 2016; Yi, Wang, et al., 2016). Various soil properties have been shown to affect emission of seed odor (Briggs & Vander Wall, 2004; Vander Wall, 1995; Yi et al., 2013). For example, scatter‐hoarding animals prefer to cache seeds in wet soil rather than in dry substrate, and seeds buried in moist soil are more likely to be pilfered by hoarding animals (Yi et al., 2013). Ash and pesticides in the soil significantly affect seed caching and cache recovery of scatter‐hoarding animals (Briggs & Vander Wall, 2004; Vander Wall, 2003). This accumulated evidence collectively indicates that the physical and chemical environments in the soil will mediate seed odor emission and consequently regulate foraging behavior of scatter‐hoarding animals under the canopy.

Korean pine (*Pinus koraiensis*) is an evergreen tree species widely distributed in northeast China and mainly relies on scatter‐hoarding animals for seed dispersal (Yi, Wang, et al., 2016). Different from other broad‐leaved species, the leaf litter of *P. koraiensis* contains a variety of volatile compounds (e.g., *α*‐pinene, *β*‐pinene, camphene, and limonene) very similar to its wingless seeds (Domrachev et al., 2012; Dormont et al., 1998; Ioannou et al., 2014; Lee et al., 2008; Yi, Li, et al., 2016; Yi, Wang, et al., 2016). Therefore, the volatile organic compounds in the leaf litter of *P. koraiensis* are expected to alter the chemical properties of forest floor and soil atmosphere under the canopy. Existing study has shown that the most abundant compounds are *α*‐ and *β*‐pinene, Δ‐3‐carene, and myrcene in the soil atmosphere under canopy of *P. koraiensis* (Smolander et al., 2006). Study by Aaltonen et al. (2011) provides further evidence that monoterpenes (*α*‐pinene, Δ3‐carene, and camphene) contribute over 90% of the emissions in the boreal pine forest floor. Recent evidence shows that the main compounds emitted by *P. koraiensis* forest floor are *α*‐pinene, Δ‐3‐carene, and camphene (Kivimäenpää et al., 2018). These floor organic compounds are volatile molecules that appear to behave similarly to seed odorants of interest to scatter‐hoarding animals. In this scenario, seed odor may be masked or even altered by forest floor scent and consequently modifies animal‐mediated seed dispersal and spatial cache placement.

Here, we hypothesized that parent‐scented forest floor facilitates disproportionate seed dispersal towards areas without conspecific trees by masking seed odor with volatile odorants and therefore modifies caching behavior of scatter‐hoarding animals. To test this hypothesis, tagged seeds were released under the canopy at the boundary between *P. koraiensis* and broad‐leaved forest to see how animal‐mediated seed dispersal was affected by volatile compounds emitted by the forest floor. In addition, we presented *P. koraiensis* seeds to food‐hoarding animals caged in artificial enclosures to test if volatiles emitted by the substrate influence seed caching and cache recovery by scatter‐hoarding animals. We expected that seeds of *P. koraiensis* would be cached under the canopy of broad‐leaved forest much more than under *P. koraiensis* covered with needle litter; and cached in substrate administrated with turpentine oil much less than in the control substrate. To our best knowledge, this is expected to be the first study to illustrate the ecological role of volatile compounds emitted by forest floor in manipulating seed dispersal by scatter‐hoarding animals and may provide further support to the mechanism maintaining the species diversity in temperate forests.

## 2 MATERIALS AND METHODS

### 2.1 Study site

Our experiments were conducted in the Qingyuan Forest Ecosystem Research Station of the Chinese Ecosystem Research Network (CERN) in the eastern Liaoning Province, Northeast China (41°51′9.94″ N, 124°56′11.22″ E, elevation 600–800 m), from September to October 2016. The climate of the region is a continental monsoon type with a humid and rainy summer and a cold and snowy winter. Mean annual air temperature varies between 3.9°C and 5.4°C with the minimum of −37.6°C in January and the maximum of 36.5°C in July. The mean annual precipitation ranges between 700 and 850 mm, 80% of which falls from June to August. The frost‐free period lasts for 130 days on average, with an early frost in October and late frost in April (Zhang & Yi, 2021). A large area of pure Korean pine *P. koraiensis* plantation forest used in this study originated from a large number of seedlings planted since 1960s (Wu & Han, 1990), which approximately covers an area of 7000 m^2^. The adjacent broad‐leaved forests covered a huge area (Figure S1), which are mainly composed of *Quercus mongolica*, *Juglans mandshurica*, *Betula platyphylla*, *Fraxinus mandshurica*, *Acer mono*, and *Phellodendron amurense*. However, *P. koraiensis* tends to mix with the broad‐leaved species at local neighborhood scales. Moreover, *P. koraiensis* can coexist with the broad‐leaved species in the natural old‐growth forest sites.

### 2.2 Soil volatiles

Soil samples were randomly collected in the central zone under the canopies of artificially planted *P. koraiensis* and the adjacent broad‐leaved understory. We collected 10–20 g surface soil (0–5 cm depth) in each of five sites apart from >20 m in each canopy for volatile analysis. Soil samples were stored at 4°C until GC–MS analysis. For each sample, 1 g soil was sealed in individual gas chromatography (GC) headspace vials (10 mL) manufactured by Daobang Technology (Nanjing, China) and kept at room temperature for 24 h prior to headspace analyses. We used the Agilent 7697A headspace autosampler coupled with Agilent 7890–5975 GC–MS (Agilent Technologies, Inc., USA) to detect volatile compounds emitted by soil. The separation of odorous components was performed by using GC connected with an HP‐17MS column (30 m length, 0.32 mm internal diameter, and 0.25 mm film thickness; Agilent Technologies, Inc., USA) (Yi, Li, et al., 2016). The following conditions were used for analysis of volatile compounds: 15 min hold at 40°C, 1°C per min to 250°C, and 10 min hold; helium carrier gas at constant flow rate of 1 mL per min. Identification of the volatiles was done according to the National Institute of Standards and Technology (NIST, Gaithersburg, MD, USA) mass spectral database. Quantification of each volatile compound identified in the headspace was termed as a percentage of total peak area.

### 2.3 Seed dispersal patterns in the field

Along the boundary line between the artificially planted *P. koraiensis* forest and the adjacent broad‐leaved forest, we established 10 seed stations (1 m × 1 m) on the ground 15 m apart from each along a single transect measuring 150 m. The transect was parallel to the boundary line between the pine and broad‐leaved forests, which will generate true replications of seed stations (Figure S1). Then, we placed 50 tagged seeds of *P. koraiensis* in each seed station. Besides the *P. koraiensis* seeds we released, *Q. mongolica* and *J. mandshurica* can also be dispersed or cached by rodents in the study area. We drilled a tiny hole measuring 0.3 mm in diameter on each seed and tethered a single plastic tag measuring 3 cm × 3.5 cm (less than 0.3 g) (Yi & Yang, 2012). Plastic tags used in this study have shown a negligible effect on seed removal by food‐hoarding animals (Kempter et al., 2018; Xiao et al., 2006). To help the experimenters locate and identify the seeds dispersed and scatter hoarded by animals, each tag was given a unique number (Yi, Li, et al., 2016). The number of seeds removed from each seed station was recorded every day for 7 consecutive days until all seeds were consumed or dispersed by rodents. At each visit, we located seeds removed by animals from each seed station with the aid of plastic tags and recorded their seed fates as intact after removal (IAR), eaten after removal (EAR), scatter hoarded (SH), and missing (M). Specifically, seeds removed from seed stations but then abandoned by rodents on the ground were recorded as IAR. Seeds removed from seed stations but then consumed by rodents on the ground were recorded as EAR. Scatter‐hoarded seeds refer to those that were buried in the surface soil or beneath leaf litter. Labeled seeds that were neither consumed at seed stations nor found on the ground surface after caching or removing were considered missing. We calculated how many seeds were scatter hoarded by animals under the canopy of *P. koraiensis* and the adjacent broad‐leaved understory, respectively. By doing so, we were able to know if scatter‐hoarding animals make less caches in the canopy of *P. koraiensis* than in the adjacent broad‐leaved understory. We failed to measure the seed dispersal distances because most of the seeds were dispersed less than 10 m away from the seed stations.

### 2.4 Capture of small rodents

In our study, SJL601 steel‐framed live traps (9 cm × 10 cm × 25 cm, manufactured by Sichuan Shujile Company, Sichuan, China) were used to trap Siberian chipmunk *Tamias sibiricus*, a dominant scatter‐hoarding animal in the study area (Yi, Li, et al., 2016). The Ethical Committee of Jiangxi Normal University (JXNU/2016025) issued permission to capture, handle, and maintain animals. The traps were pre‐baited for 1 day with carrots and peanuts and set at 0800 hrs in forests 3 km away from the study area. We wrapped all traps with steel mesh to protect the trapped animals from predators and placed them at 5‐m intervals along four transects. Because *T. sibiricus* have two daily activity peaks, we checked the traps twice every day (at 11:00 h and 16:00 h, respectively). Upon capture, adult *T. sibiricus* were immediately transported to the animal housing room and raised individually in medium‐sized cages (30 cm × 40 cm × 50 cm) provided with nests, peanuts, pine seeds, and water ad libitum. However, other rodents (e.g., *Sciurus vulgaris*, *Apodemus peninsulae*, and *Myodes rufocanus*) were immediately released from the traps because they were either rarely distributed or were predominant larder hoarders. All cages provided with bedding material and building blocks were kept at a natural photoperiod (about 14 light hours) and a room temperature schedule (day 15–20°C, night 10–15°C). They were provided with tap water and rat chow (Zhongke Scientific and Technical Company, Guangzhou, China) ad libitum. To ensure animal welfare, we strictly followed the Regulations for Experimental Animals issued by China's State Council in January 1988, which were put into effect by China's State Scientific and Technological Commission in November 1988 (Yi, Li, et al., 2016). We released *T. sibiricus* to the forests where they were captured after the seed‐hoarding experiments in the enclosures.

### 2.5 Seed hoarding by small rodents in artificial enclosures

After 1‐week acclimation indoors, *T. sibiricus* were introduced into enclosures for scatter hoarding and cache recovery. Seed‐hoarding experiments were carried out in three identical artificial enclosures (10 m × 10 m × 2 m) established near the canopy (see more details in Yi, Steele, et al., 2016). The floor of each enclosure was paved uniformly with red bricks. We then carefully removed 64 bricks to create 64 evenly spaced, small, shallow pits (length × width × depth: 24 cm × 12 cm × 6 cm). The pits were separated by 1 m and formed an 8 × 8 grid. We used fine sand to fill the pits in each enclosure, providing locations and substrates for caching by *T. sibiricus*. This deployment provided an idea arena for us to investigate seed caching only in the small pits by *T. sibiricus* (Yi, Steele, et al., 2016). To test the effects of substrate odor on the seed scatter‐hoarding behaviors of *T. sibiricus*, 32 pits were randomly selected in each enclosure and several drips (less than 0.2 mL) of turpentine oil (Dingshinxin Chemical Co. Ltd., Tianjin, China) were administrated into each pit, which has been pre‐tested several times to ensure that the amount of turpentine oil is not too much to deter the rodents. We added turpentine oil to simulate the forest floor of *P. koraiensis* and then masked seed odor because it is mainly composed of *α*‐ and *β*‐pinene (Mercier et al., 2009). However, the remaining 32 pits were turpentine oil free and treated as control group in each enclosure. Immediately after 60 *P. koraiensis* seeds were placed in the seed station established at the center of each enclosure in the morning (08:00 h), one individual *T. sibiricus* was introduced into each enclosure to forage and cache freely for several hours. Each *T. sibiricus* was trapped and transferred to the housing facility at 16:00 h. Then, all 64 pits in each enclosure were carefully checked to see how many caches were established and how many seeds were cached in the control or turpentine oil‐applied pits, respectively. According to the previous study (Yi, Steele, et al., 2016), we grouped seed fates into IIS, EIS, EAR, IAR, SH, and LH, which stand for intact in situ (seeds remained intact in seed stations), eaten in situ (seeds were eaten in seed stations), eaten after removal (seeds were eaten in other places), intact after removal (seeds were removed from seed stations and abandoned on the floor of enclosures), scatter hoarded (seeds were buried in the shallow pits), and larder hoarded (seeds stored in the artificial nests), respectively. We used four males and six females to test our predictions. To minimize observer bias, blinded methods were used when all behavioral data were recorded and/or analyzed.

### 2.6 Cache recovery by small rodents in the enclosures

To test how the volatiles applied in the substrate affected cache recovery by *T. sibiricus*, we buried one seed 1 cm deep in each of the 64 pits in each enclosure at 08:00 h. The turpentine oil‐applied pits were the same as in the previous experiment because the volatiles can last several days. Then, one individual of *T. sibiricus* was immediately introduced into each enclosure and allowed to recover the artificial caches for 8 h. At 16:00 h, we checked the difference in cache recovery rates between the control and the turpentine oil‐treated pits. The same 10 individual chipmunks (4♂and 6♀) were tested.

### 2.7 Data analyses

The Statistical Package for Social Sciences (SPSS) Version 16.0 was used for data analyses in our study. Independent t test was applied to comparison of volatile compounds of soil beneath the canopy between the conspecific and interspecific forests. The Mann–Whitney' test of was used to examine differences in the proportion of seeds scatter hoarded or eaten after removal between the conspecific and interspecific canopy. We used paired samples *t* test to detect difference in scatter hoarding, cache recovery rates, and cache number in the artificial enclosures because we randomly selected half of the shallow pits to administrate turpentine oil. The amounts of seed cached and eaten in this study were the average number of seeds unless mentioned elsewhere. *p* < .05 was considered statistically significant.

## 3 RESULTS

**FIGURE 1:**
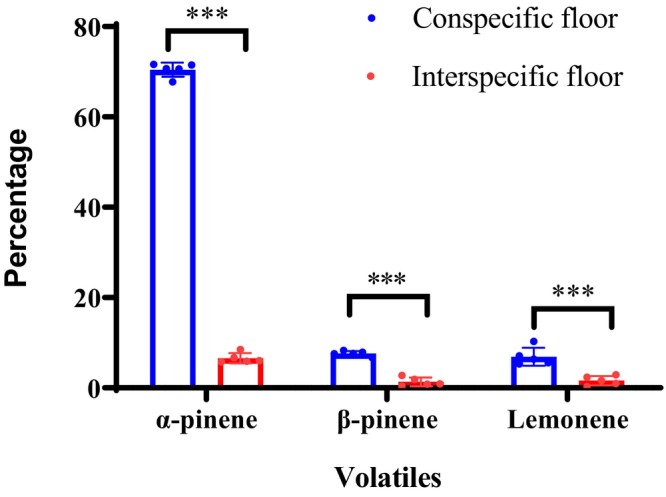
The main volatile compound emitted by *P. koraiensis* floor and the adjacent broad‐leaved forest floor. *** stands for *p* < .001.

**FIGURE 2:**
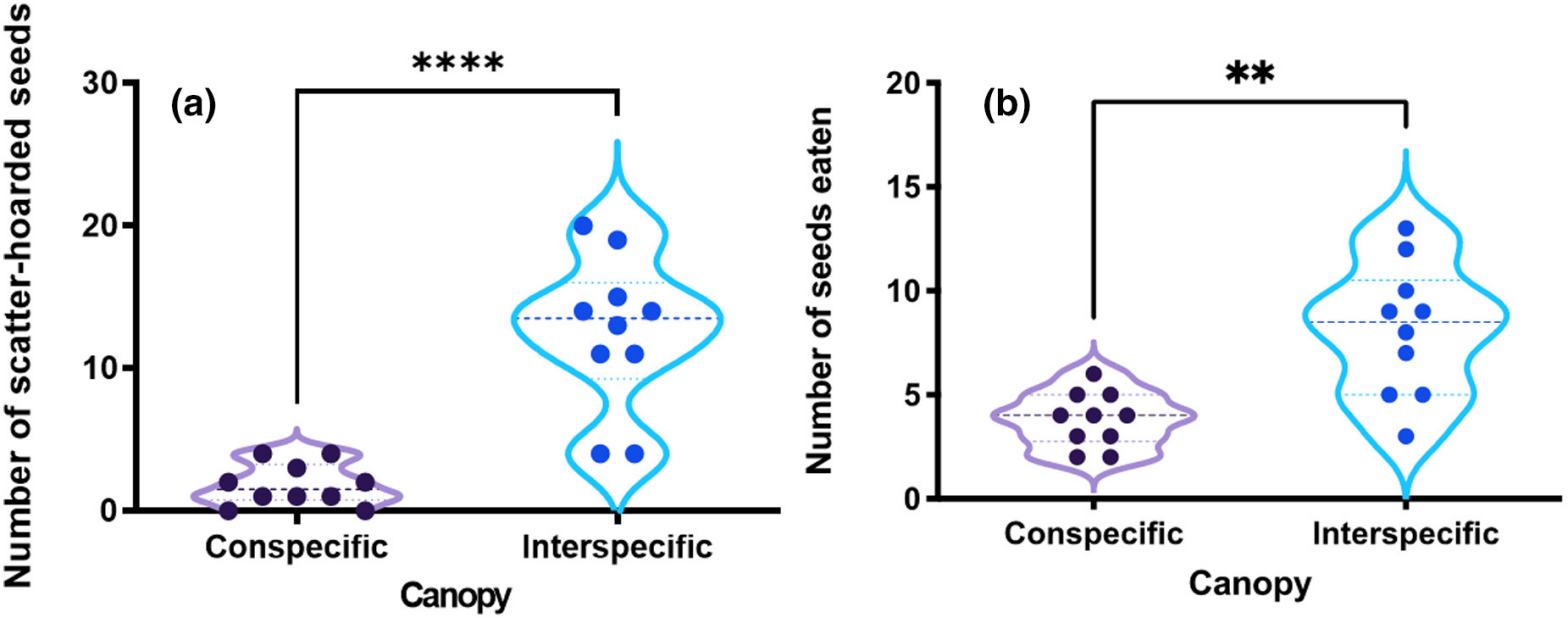
Seeds eaten (a) and scatter hoarded (b) by food‐hoarding animals in the conspecific and interspecific areas. ** and **** represent *p* < .01, and .0001, respectively.

The GC–MS analyses showed that *P. koraiensis* forest floor emitted more volatiles than the adjacent broad‐leaved forest floor in terms of *α*‐pinene, *β*‐pinene, and limonene (*t*_1,8_ = 73.87, *p* < .001; *t*_1,8_ = 12.24, *p* < .001; *t*_1,8_ = 5.24, *p* < .001; Figure 1). Our field observations clearly showed that scatter‐hoarding animals moved and ate more *P. koraiensis* seeds in the adjacent broad‐leaved understory than in the conspecific canopy (*t* = 4.502, df = 9, *p* = .002; Figure 2a). As expected, more seeds of *P. koraiensis* were scatter hoarded toward the adjacent broad‐leaved understory rather than the conspecific canopy of *P. koraiensis* (*t* = 6.751, df = 9, *p* < .001; Figure 2b).

**FIGURE 3:**
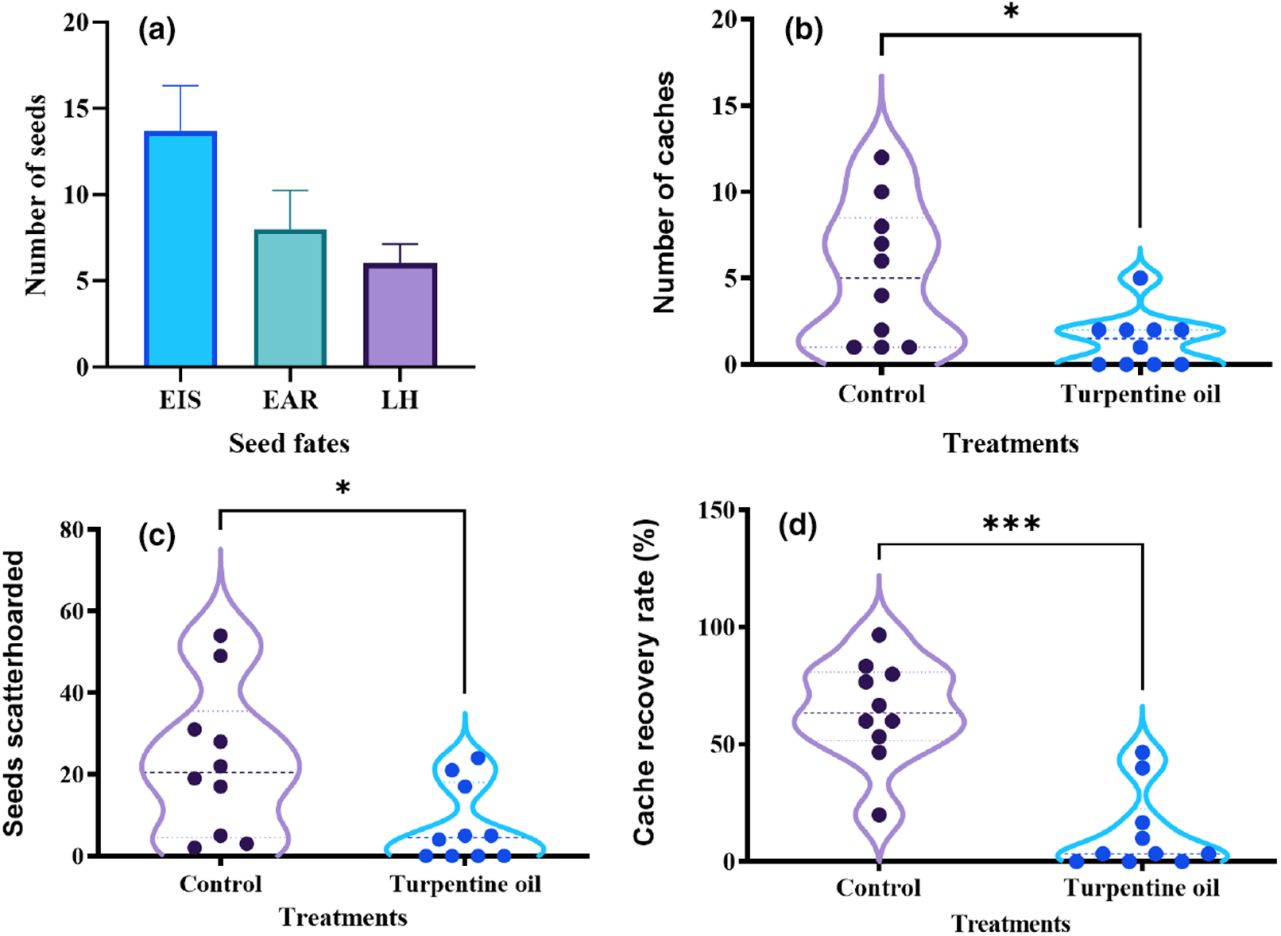
The effects of substrate on seed dispersal (a), scatter hoarding (b and c), and cache recovery (d) by *Tamias sibiricus*. * and *** represent *p* < .05, and .001, respectively.

We found that 22.8%, 13.3%, and 10.0% of seeds released were eaten in situ, eaten after removal, and larder hoarded by the chipmunks, respectively (Figure 3a). Experiments in the enclosures provided evidence that scatter‐hoarding animal *T. sibiricus* preferred to cache seeds of *P. koraiensis* into the turpentine oil‐free pits rather than the pits applied with turpentine oil, as indicated by the number of caches and buried seeds, respectively (*t*_1,9_ = −2.890, *p* = .018; *t*_1,9_ = −2.316, *p* = .046; Figure 3b,c). Compared to seeds larder hoarded, 51.0% of seeds released were scatter hoarded by the chipmunks. Moreover, seeds of *P. koraiensis* buried in the turpentine oil‐treated pits were less likely to be recovered than in the control pits by *T. sibiricus* (*t*_1,9_ = −4.811, *p* = .001; Figure 3d).

## 4 DISCUSSION

Similar to the previous studies (Aaltonen et al., 2011; Kivimäenpää et al., 2018; Smolander et al., 2006), volatile compounds emitted by the forest floor of artificially planted *P. koraiensis* in northeast China are mainly composed of *α*‐pinene, *β*‐pinene, and limonene in our study. These compounds are primarily synthesized in the needles of *P. koraiensis* and delivered to the forest floor by defoliation. However, *α*‐pinene, *β*‐pinene, and limonene in soil are negligible in the adjacent broad‐leaved forest floor compared to the *P. koraiensis* forest floor, indicating that leachate of the foliar litters is the main source of volatile compounds under the canopy of *P. koraiensis* in our study. Moreover, we provide first evidence that the huge differences in surface volatiles between the pine and broad‐leaved canopies are responsible for cache site selection by food‐hoarding rodents, which cause disproportionate deposition in spatially heterogeneous environments.

Previous studies have accumulated that volatile compounds emitted by *P. koraiensis* seeds are α‐pinene, β‐pinene, limonene, and camphene (Yi, Li, et al., 2016; Yi, Wang, et al., 2016), which match well with the volatile profile of the forest floor of *P. koraiensis*. Therefore, volatile odorants emitted from seeds are speculated to be masked or concealed by the scent emitted by the forest floor of *P. koraiensis*. Scatter‐hoarding rodents have been suggested to partially rely on olfaction as a foraging strategy (Vander Wall, 1990). In this scenario, recovery of the buried seeds in soil by scatter‐hoarding animals will be more difficult under the canopy of *P. koraiensis* than the adjacent broad‐leaved forest floor, where seed odor is supposed to be more contrasting against the forest floor. As expected, scatter‐hoarding animals avoided to scatter‐hoard seeds in the canopy of *P. koraiensis* but selectively transport seeds of *P. koraiensis* toward the adjacent broad‐leaved understory for caching. Despite the importance of floor scent in directing seed dispersal, we are unable to rule out the possibility of conspecific competition, cache pilferage risk, and predation risk in driving food‐hoarding animals to move seeds further away from the mother trees for hoarding (Cao et al., 2018; Steele et al., 2014, 2015; Sun & Zhang, 2013). Scatter‐hoarding animals caching *P. koraiensis* seeds in the adjacent broad‐leaved understory not only facilitates cache recovery due to the prominent seed odor but may also reduce spatial memory input on their caches because scatter‐hoarding animals place less memory on caches emitting strong odor (Li et al., 2018). Some may argue that caching seeds on the floor of *P. koraiensis* will decrease the possibility of cache pilferage, however, it will also render low cache recovery rate given the forest floor volatiles behave similarly to seed odor. Although caches in the adjacent broad‐leaved understory will be at high risk of cache pilferage, accurate spatial memory of scatter‐hoarding animals will compensate cache loss to some degree. Therefore, caching seeds by scatter‐hoarding animals in the adjacent broad‐leaved understory rather than in the canopy of *P. koraiensis* may guarantee more rewards from their own caches, reflecting a trade‐off between maximizing cache recovery and minimizing cache loss.

We admit that 1 year's field data will not necessarily validate our findings. However, our behavioral experiments in the enclosures, consistent with the results in the field, showed that scatter‐hoarding animal *T. sibiricus* established more caches of *P. koraiensis* in the control pits than in the turpentine oil‐applied pits in the enclosures. Although 10% of seeds released were larder hoarded, scatter‐hoarding rodent *T. sibiricus* consistently avoid the area with scent of mother trees to establish their scattered caches. More seeds cached in the control pits indicated that *T. sibiricus* selectively avoided the turpentine oil‐applied pits to establish their caches. Previous studies have shown that chemical properties of substrate significantly affected cache site selection of scatter‐hoarding animals (Downs & Vander Wall, 2009; Yi et al., 2013). The observed patterns of cache recovery both in the control and the turpentine oil‐applied pits suggest that volatile compounds concealed seed odor of *P. koraiensis*, hampering olfaction by *T. sibiricus*, and consequently decreasing the rate of cache recovery. The fact that less cached seeds were recovered from the odor‐masked pits leads us to better understand the role of cache recovery in determining cache site selection. Odorant match between seeds and floor substrate significantly modified seed dispersal and spatial cache placement mediated by food‐hoarding animals. Combined with the results in the field, we suggest that *P. koraiensis* and possibly other conifer species (e.g., cypress and fir) may promote disproportionate seed dispersal into interspecific areas exhibiting contrasting floor volatile profiles. Our results may also have implications for other broad‐leaved species that emit volatile organic compounds manipulating scatter‐hoarding behavior of rodents.

*Pinus koraiensis*, widely distributed in far east Asia, usually forms sparse forest floor although sunlight intensity under the forests is sufficient for plants to grow, reflecting the strong inhibitory activity of leaf litter leachates on seed germination and seedling growth in the sparse forest floors. Even seedlings and siblings of *P. koraiensis* are seldom found under its own canopy, suggesting that litter leachates of *P. koraiensis* may hinder its own seed germination and seedling growth via the release of allelochemicals into the forest floor (Chen et al., 2016). Therefore, scatter‐hoarding animals dispersing seeds away from parent tree and directly caching into the broad‐leaved understory are expected to benefit seed germination and seedling establishment. In our study, non‐random seed dispersal into the interspecific areas is likely to result from the caching behavior of food‐hoarding animals which prefer the areas where there is a mismatch between seed odor and floor scent. Given that disproportionate seed dispersal into the adjacent broad‐leaved understory is beneficial for escaping inhibitory of litter leachate near the parent tree and colonization of vacant sites, parent‐scented floor masking seed odor is expected to promote seed transportation and seedling colonization into new habitats, conferring a pronounced advantage to seed dispersal of *P. koraiensis*. Pines bearing seeds emitting volatile compounds similar to those of foliar litters highlight the role of forest floor chemistry in directing seed dispersal and shifting pine–rodent conditional mutualism toward more mutualistic. Seed dispersal studies have primarily examined the mechanisms by which plants influence dispersal immediately around the parent plant by seed dispersers (Deniau et al., 2017; Freckleton & Lewis, 2006; Packer & Clay, 2003; Pregitzer et al., 2010); our study provides insight into an alternative mechanism by which *P. koraiensis* increase selection for seed dispersal away from the parent trees, where they may be particularly vulnerable to mortality.

Although we focused on distance‐dependent seed dispersal by vertebrates without evaluating seed and seedling survival, distance‐dependent seedling mortality caused by vertebrate predators has been well observed in pines and other broad‐leaved species (Bayandala & Seiwa, 2016; Finkel, 2011; Holík & Janík, 2022; Qin et al., 2020; Yamazaki et al., 2009). Therefore, our study may represent a distance‐dependent mechanism of seed dispersal, which can potentially contribute to the Janzen–Connell effects. Our findings highlight the importance of scatter‐hoarding rodents for distance‐dependent seed dispersal beneath conspecific and adjacent heterospecific canopy. Our study may also shed light on the role of forest floor chemistry in manipulating tree–rodent interactions that facilitate species coexistence and formation of mixed forests. We recommend future studies focusing on animal‐mediated seed dispersal pay more attention to the effects of substrate property of forests on regulating plant–animal interactions and seed dispersal effectiveness, which is important for forest conservation and the maintenance of biodiversity.

## AUTHOR CONTRIBUTIONS

**Shuhua Guo:** Data curation (equal); investigation (equal); writing – original draft (equal). **Xianfeng Yi:** Data curation (equal); formal analysis (equal); writing – review and editing (equal). **Meixia Sui:** Conceptualization (equal); supervision (equal); writing – review and editing (equal).

## CONFLICT OF INTEREST STATEMENT

The authors declare no competing interests.

## Supporting information


Figure S1.

## Data Availability

Data used in this study will be available at Figshare (https://doi.org/10.6084/m9.figshare.25151453).
