# Supplementary material for: Substrate scent‐induced disproportionate seed dispersal by rodents
Source: Ecol Evol. 2024 Jul 21;14(7):e70075. doi: 10.1002/ece3.70075 (PMC11260879; doi:10.1002/ece3.70075)

 Fig. S1. Study site and experimental design. Yellow dots represent seed stations on the transect. At the left of the transect is artificially planted Korean pine forest, and right the broad-leaved forest.


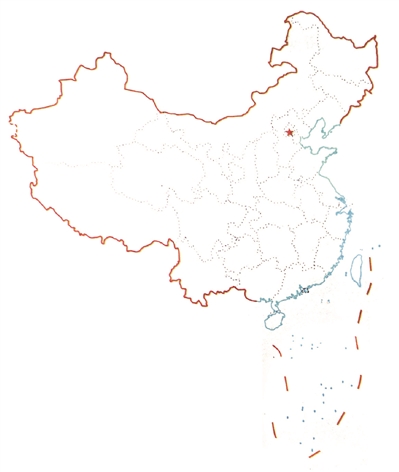

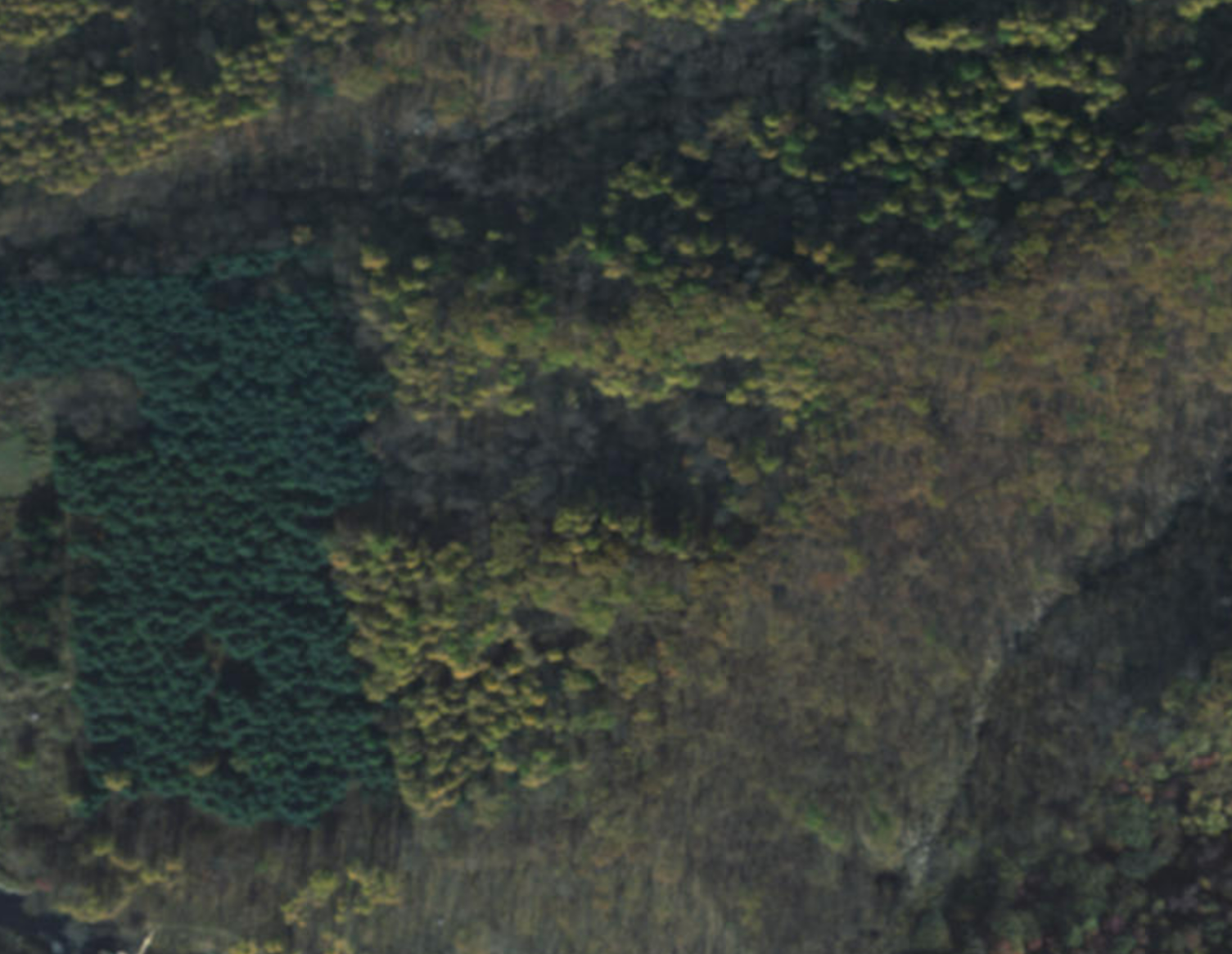

Supplement: Supplementary file 1 — Figure S1. [file ECE3-14-e70075-s001.docx]
